# Traumatic Haemorrhagic Cervical Lymphadenopathy with Underlying Infectious Mononucleosis

**DOI:** 10.1155/2017/3097414

**Published:** 2017-10-18

**Authors:** George Rahmani, Sarah Power

**Affiliations:** Department of Radiology, Beaumont Hospital, Dublin, Ireland

## Abstract

A 16-year-old male presented to the Emergency Department with a painful 3 × 3 cm left-sided neck swelling six hours following blunt trauma to the neck from a heavy swinging door. A CT angiogram was performed which revealed a large haemorrhagic lymph node as well as generalised cervical lymphadenopathy. The patient was eventually diagnosed with infectious mononucleosis. This case report describes a rare case of traumatic haemorrhagic cervical lymphadenopathy with an underlying diagnosis of infectious mononucleosis.

## 1. Introduction

Patients with infectious mononucleosis most commonly present with a triad of fever, lymphadenopathy, and pharyngitis [1]. Splenomegaly can also occur and these patients have an increased risk of traumatic splenic rupture [2]. Lymphadenopathy as a result of trauma is quite rare with only a handful of case reports in the literature [3, 4]. Haemorrhagic lymphadenopathy classically occurs in the core of necrotic lymph nodes that are affected by malignancy, either primary malignancy, as in lymphoma, or secondary metastasis [5]. Haemorrhagic lymphadenopathy has also been described in other conditions such as primary al amyloidosis [6]. We describe a case of haemorrhagic cervical lymphadenopathy following blunt force trauma to the neck in a patient with an underlying diagnosis of infectious mononucleosis.

## 2. Case

**Figure 1:**
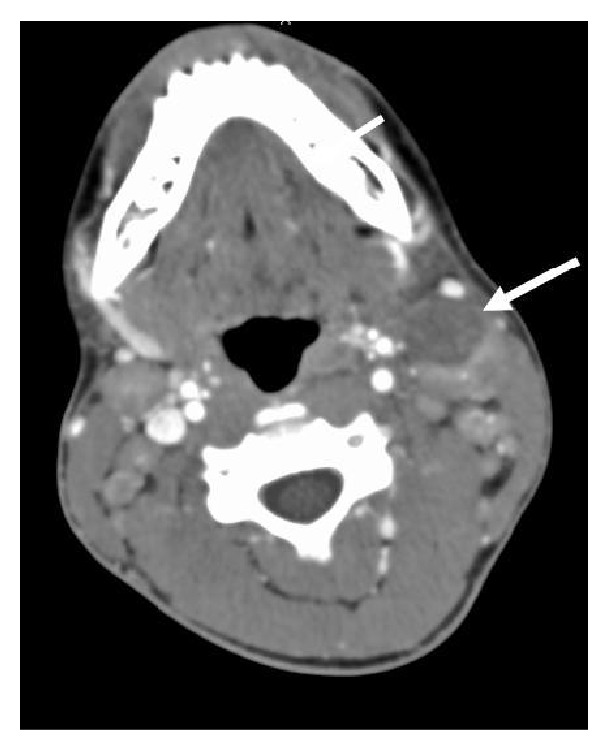
Axial CT angiogram showing bilateral cervical lymphadenopathy, the largest of which measures 4 × 3.4 × 2.5 cm (arrow). The posterior portion of the node demonstrates arterial phase enhancement while the anterior portion does not demonstrate it. This mixed density mass has the appearance of a necrotic or haemorrhagic lymph node.

**Figure 2:**
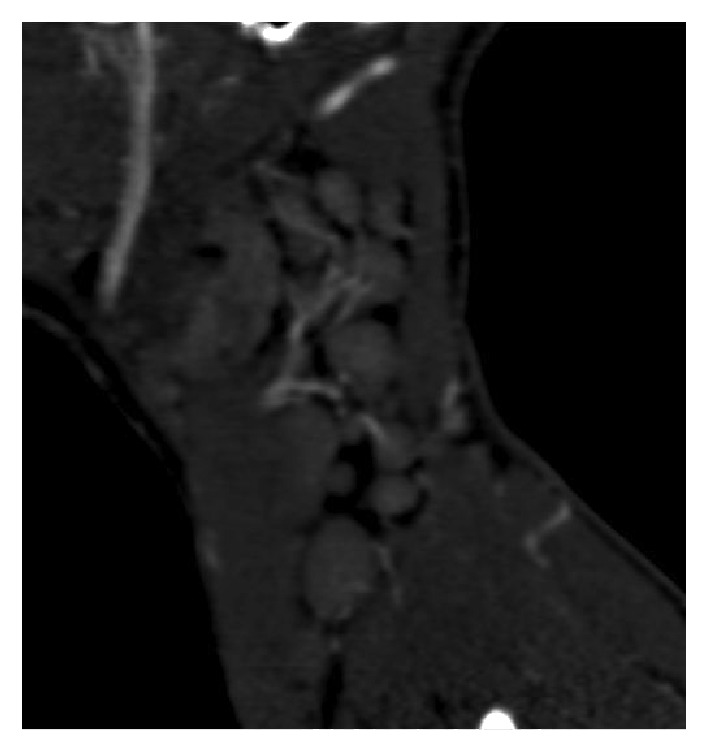
Sagittal CT angiogram demonstrating extensive hypervascularity of lymph nodes in the left neck.

**Figure 3:**
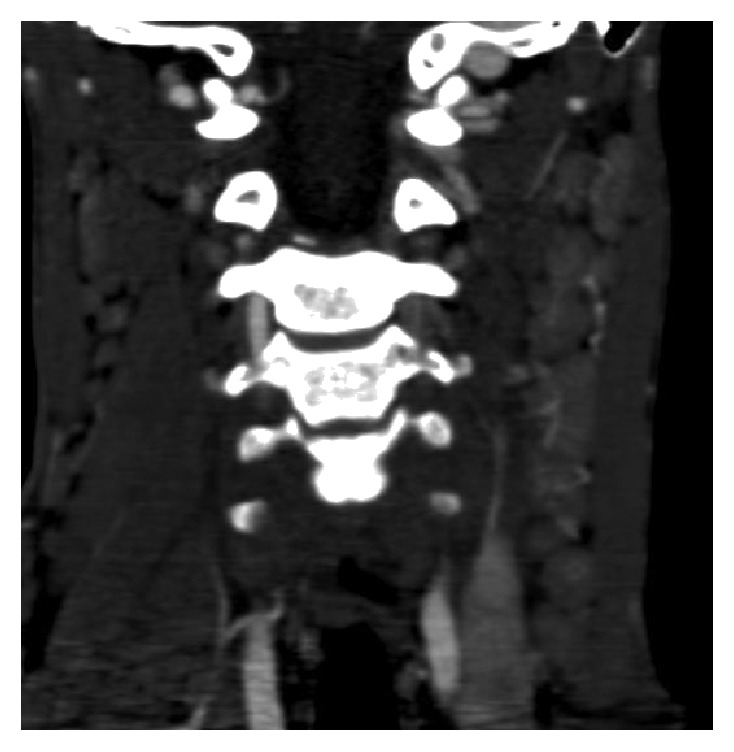
Coronal CT angiogram of the neck demonstrating extensive hypervascularity of cervical lymph nodes bilaterally which are more pronounced on the left side.

A 16-year-old male presented to the Emergency Department with a painful 3 × 3 cm left-sided neck swelling six hours following blunt trauma to the neck from a heavy swinging door. His vital signs were normal and he was alert and orientated in time, place, and person. His neurological examination was unremarkable. The swelling was nonpulsatile, but given the history of rapid growth, a computed tomography (CT) angiogram was performed. It did not demonstrate any vascular injury but revealed significant bilateral cervical lymphadenopathy, largest on the left side, level II measuring 4 × 3.4 × 2.5 cm, with mixed density suggestive of either a necrotic lymph node or, given the history of trauma and rapid swelling, haemorrhage into the lymph node (Figure 1). Of note, the lymphadenopathy showed signs of hypervascularity which was more in keeping with an infectious aetiology (Figures 2 and 3). The patient denied symptoms of weight loss or night sweats but complained of minor fatigue in recent weeks.

**Figure 4:**
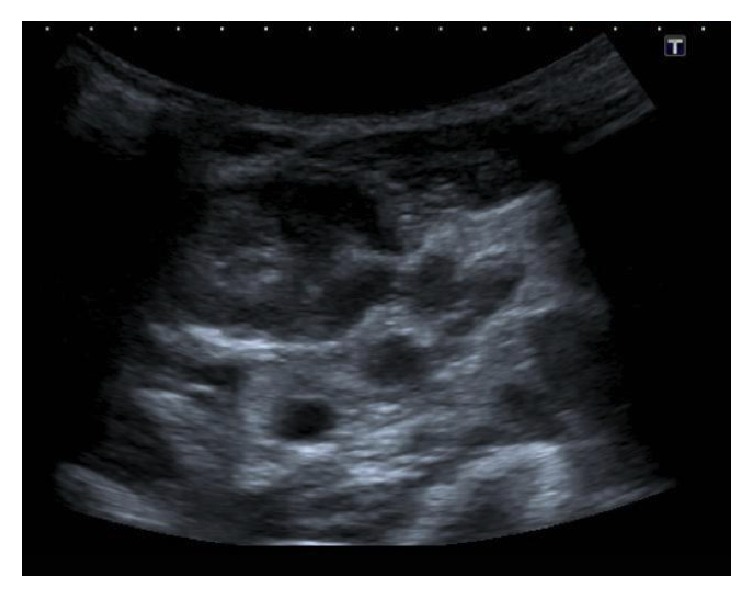
Ultrasound scan showing an enlarged left cervical lymph node with heterogeneous echogenicity.

**Figure 5:**
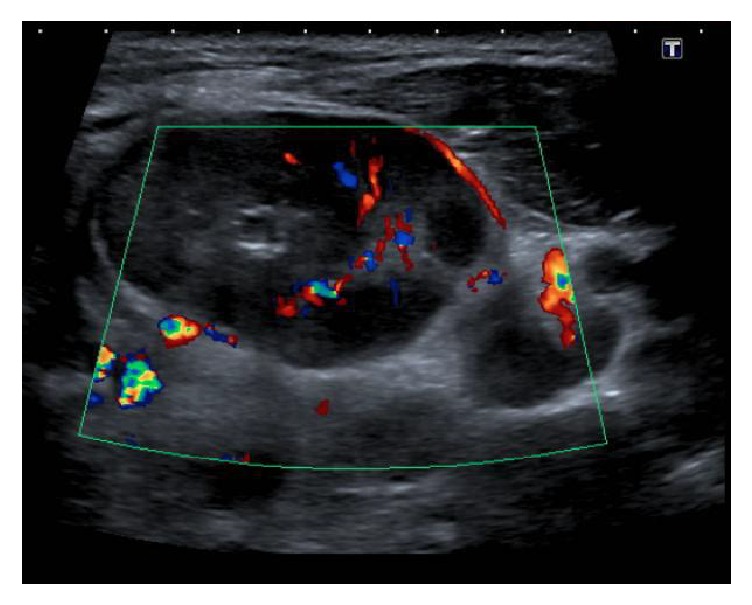
Ultrasound scan of the lesion in question showing colour Doppler flow in the posterior aspect of the lesion only.

**Figure 6:**
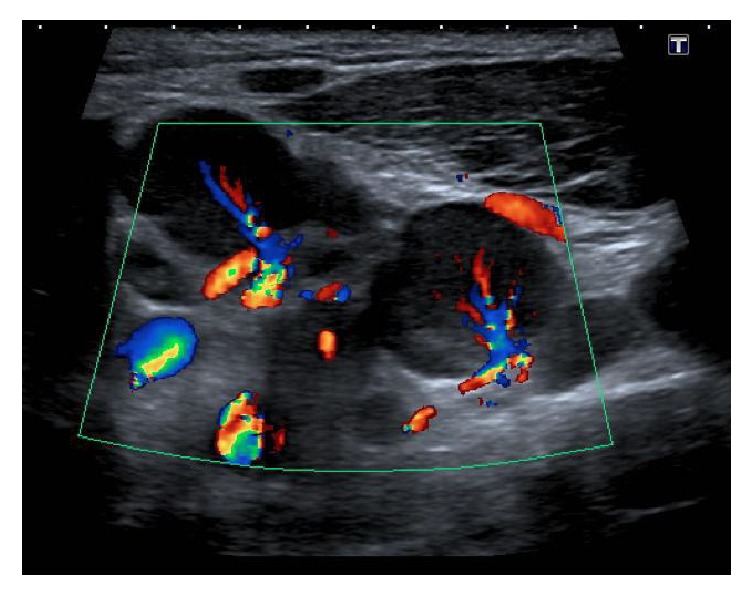
Ultrasound scan showing increased colour Doppler flow through enlarged left cervical lymph nodes.

An ultrasound scan was performed to further characterise the lymphadenopathy, which demonstrated heterogeneous internal echoes with absent colour Doppler flow in the anterior aspect of the lesion (Figures 4 and 5). Ultrasonography also demonstrated generalised hypervascular cervical lymphadenopathy, again reflective of an infectious aetiology (Figure 6). A CT thorax did not demonstrate lymphadenopathy elsewhere.

A full blood count revealed a mild leucocytosis (12.8 × 10^9^; normal range 4–11 × 10^9^) with elevated monocytes (1.26 × 10^9^; normal range 0.2–1.0 × 10^9^). Of note, the lymphocyte count of 3.83 × 10^9^ was within normal limits (1.00–4.00 × 10^9^) and additional microscopy did not demonstrate a disproportionate increase in atypical lymphocytes. The patient's coagulation profile was also normal.

A monospot test was positive for the presence of heterophile antibodies and further serological testing was carried out which detected the presence of IgM and IgG antibodies to the Epstein-Barr viral capsid antigen (VCA). Serological testing was negative for* Cytomegalovirus*,* Toxoplasma gondii*,* Treponema pallidum*, and hepatitis B and hepatitis C. Testing for IgG to Epstein-Barr nuclear antigen (EBNA) was also negative which helped to rule out a previous EBV infection.

In light of positive EBV serology and generalised hypervascular cervical lymphadenopathy, a diagnosis of infectious mononucleosis was made. Given the recent history of trauma, the left-sided neck swelling was determined to be a haemorrhagic lymph node. To the best of our knowledge, this is the first report of traumatic haemorrhagic cervical lymphadenopathy with an underlying diagnosis of infectious mononucleosis.

## 3. Discussion

Infectious mononucleosis, commonly referred to as “glandular fever,” is one of the most common causes of prolonged illness in young adults. It is the manifestation of primary Epstein-Barr virus infection, which is latent in approximately 90% of the population [1]. The vast majority of people that have been exposed to EBV are asymptomatic. Approximately one-third of young adults will develop symptoms, of which the triad of fever, lymphadenopathy, and pharyngitis is found in 50% of cases [7, 8]. Other physical manifestations of infectious mononucleosis include splenomegaly, hepatomegaly, or palatal petechiae, which are each present in approximately 10% of cases [1]. The diagnosis is confirmed by serology; the presence of heterophile antibody is used as a rapid screening test with a sensitivity of 70–90% and specificity of 96–99% with rare false positives occurring in conditions such as viral hepatitis and* Cytomegalovirus* infection [9]. Testing for the presence of IgG and IgM antibodies to the Epstein-Barr VCA is more sensitive and specific for infectious mononucleosis and these antibodies are usually present by the time the disease has manifested clinically. Anti-VCA IgM remains in the circulation for approximately 1-2 months, while anti-VCA IgG will persist for life. The presence of IgG to EBNA suggests past EBV infection and in the context of positive serology is suggestive of reactivation of latent EBV.

Proliferation of mononuclear cells causes lymphadenopathy associated with infectious mononucleosis and can mimic other more serious underlying conditions, such as lymphoma. In some rare cases, these lymph nodes have been reported to contain geographic areas of necrosis and infarction [3, 10]. With regard to trauma, a case of lymph node infarction due to chronic trauma in the knee of a healthy patient has been previously described, mimicking synovial sarcoma on MRI [4]. In this case, the patient did not have underlying lymphadenopathy, and there was a history of chronic minor traumas, rather than an acute precipitory event.

The differential diagnosis for cervical lymphadenopathy would include other infectious aetiologies including viruses such as cytomegalovirus and herpes simplex virus, bacterial infections and mycobacterium infections like tuberculosis, and* Mycobacterium avium*-intracellulare complex infections. Malignancy is an important differential that must be ruled out including lymphoma and metastases from head and neck tumours. Other neoplastic lesions can cause cervical lymphadenopathy including Castleman disease and Kaposi sarcoma.

In our case, the appearance of the lymph node on CT and ultrasound was that of a heterogeneous soft tissue mass with absence of blood flow/contrast enhancement in the anterior portion. The differential diagnosis for the appearance of such a lesion was that of a necrotic or haemorrhagic lymph node. While cases of lymph node necrosis have been previously described in cases of infectious mononucleosis, given the acute history of trauma and the rapid appearance of the swelling, traumatic haemorrhage into the underlying abnormal lymph node is a more likely diagnosis. It may be that the mechanism of such an injury is likened to traumatic splenic rupture in the context of splenomegaly in response to EBV infection [2]. To the best of our knowledge, this is the first report of traumatic haemorrhagic cervical lymphadenopathy leading to an underlying diagnosis of infectious mononucleosis.
